# Role of Damage Associated Molecular Pattern Molecules (DAMPs) in Aneurysmal Subarachnoid Hemorrhage (aSAH)

**DOI:** 10.3390/ijms19072035

**Published:** 2018-07-13

**Authors:** Shafqat Rasul Chaudhry, Ahmad Hafez, Behnam Rezai Jahromi, Thomas Mehari Kinfe, Alf Lamprecht, Mika Niemelä, Sajjad Muhammad

**Affiliations:** 1 Department of Neurosurgery, University Hospital Bonn, University of Bonn, Sigmund-Freud Str. 25, D-53105 Bonn, Germany; shafqatrasul@yahoo.com (S.R.C.); Thomas.Kinfe@ukb.uni-bonn.de (T.M.K.); 2 Department of Neurosurgery, University of Helsinki and Helsinki University Hospital, 266, FI-00029 Helsinki, Finland; ext-ahmad.hafez@hus.fi (A.H.); behnam.rezai-jahromi@hus.fi (B.R.J.); mika.niemela@hus.fi (M.N.); 3 Department of Pharmaceutics, Institute of Pharmacy, University of Bonn, Gerhard-Domagk-Strasse 3, D-53121 Bonn, Germany; alf.lamprecht@uni-bonn.de

**Keywords:** subarachnoid hemorrhage, danger associated molecular pattern molecules (DAMPs), alarmins, high mobility group box-1 (HMGB1), S100B, mitochondrial DNA, hemoglobin, interleukin (IL)-33, IL-1α, heat shock proteins

## Abstract

Aneurysmal subarachnoid hemorrhage (aSAH) represents only a small portion of all strokes, but accounts for almost half of the deaths caused by stroke worldwide. Neurosurgical clipping and endovascular coiling can successfully obliterate the bleeding aneurysms, but ensuing complications such as cerebral vasospasm, acute and chronic hydrocephalus, seizures, cortical spreading depression, delayed ischemic neurological deficits, and delayed cerebral ischemia lead to poor clinical outcomes. The mechanisms leading to these complications are complex and poorly understood. Early brain injury resulting from transient global ischemia can release molecules that may be critical to initiate and sustain inflammatory response. Hence, the events during early brain injury can influence the occurrence of delayed brain injury. Since the damage associated molecular pattern molecules (DAMPs) might be the initiators of inflammation in the pathophysiology of aSAH, so the aim of this review is to highlight their role in the context of aSAH from diagnostic, prognostic, therapeutic, and drug therapy monitoring perspectives. DAMPs represent a diverse and a heterogenous group of molecules derived from different compartments of cells upon injury. Here, we have reviewed the most important DAMPs molecules including high mobility group box-1 (HMGB1), S100B, hemoglobin and its derivatives, extracellular matrix components, IL-1α, IL-33, and mitochondrial DNA in the context of aSAH and their role in post-aSAH complications and clinical outcome after aSAH.

## 1. Introduction

Aneurysms are the weak bulging lesions or abnormal dilatations present in 1–5% of the adult population [1]. Aneurysms are formed due to the hemodynamic shear stress in the arterial wall at the bifurcation of arteries and are marked by chronic inflammation and degeneration in the arterial wall [2,3]. The rupture of an intracranial aneurysm leads to subarachnoid hemorrhage that accounts for only 5% of all the stroke events, but the mortality inflicted by it is around 50% (32 to 67%) and affects a relatively younger age group compared to ischemic stroke [4,5,6]. The incidence of aSAH is estimated around 10.5 per 100,000 persons per year, but varies geographically with higher incidence in Japan (22.7) [4,7]. The fatality of the disease is reflected by 20% deaths occurring before any medical attention, 30% within 24 h of onset, and 40–60% within a month after subarachnoid hemorrhage [4,8]. Among the survivors, one third remains lifelong dependent and those who have a good recovery still have neurological and/or cognitive deficits [9,10].

**Figure 1:**
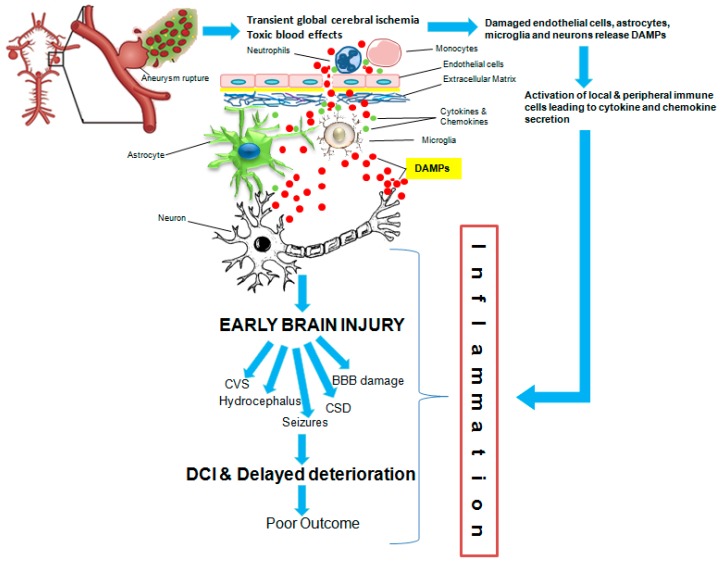
Schematic representation of the role of inflammation mediated by DAMPs and cytokines released after aSAH and their association with post-aSAH complications and clinical outcome.

The obliteration of the bleeding aneurysm from the arterial circulation is achieved by neurosurgical clipping and endovascular coiling in the majority of cases [11], but still, the outcome for the patients is devastating. This is mainly due to the post-aSAH complications occurring mainly over the first two weeks after initial bleeding [12]. The brain injury following aSAH occurs in two phases. An early brain injury occurring within initial 72 h of the insult results from transient global ischemia and toxic effects of the extravasated blood [11,12,13]. This may be followed by a secondary delayed phase of brain damage over a period of 3–14 days and is the time frame where post-aSAH complications can develop and cause neurological deterioration [12]. The major post-aSAH complications include rebleeding, cerebral vasospasm (CVS), hydrocephalus, seizures, delayed ischemic neurological deficits (DIND), cortical spreading depression, delayed cerebral ischemia (DCI), infections, cardiomyopathy, and pulmonary edema [9]. The research in the past was maligned by a sole focus on cerebral vasospasm and strategies aimed at its reversal were developed. The failure of the endothelin antagonists to improve the outcome despite reversing the cerebral vasospasm has recognized that the clinical outcome after aSAH is determined by multiple factors. These conflicting results led to changes in the direction of aSAH research to early brain injury with profound stress laid on the role of inflammation [5] that plays crucial and central role during the development of post-aSAH complications. Inflammation occurring in the absence of pathogens (as in case of aSAH) is usually ascribed as sterile inflammation, however, involves similar cascades of mechanisms mounted against pathogens [14]. This is owing to the pattern recognition receptors (PRRs) which respond to the evolutionarily conserved danger molecular motifs, which may either be exogenous ‘pathogen associated molecular patterns (PAMPs)’ derived from pathogens or endogenous ‘DAMPs’ molecules derived from injured, stressed, and necrotic cells [14,15,16]. Immediately after acute brain injury, local and systemic inflammatory response leads to trigger inflammatory signaling cascades accompanied by the activation and infiltration of immune cells at the site of injury [17]. A great body of evidence supports the critical role of inflammation in the aSAH [18,19] (Figure 1).

Any type of injury, either ischemic or traumatic, can potentially release DAMPs from injured or stressed cells leading to inflammation without the presence of any pathogens. During sterile inflammation DAMPs bind to the PRRs on immune cells, leading to the activation of subcellular signaling pathways including Nuclear Factor-κB (NFκB) and finally, upregulates the expression of multiple genes including the transcription and release of pro-inflammatory mediators [14,16,20]. Over the past years, an ever expanding list of DAMPs, along with their cognate receptors, has been identified including HMGB1, HSPs, S100 proteins, SAP130, ATP, mitochondrial DNA, formyl peptides, heparin sulphate, β-amyloid, biglycan, versican, IL-1α, IL-33, cholesterol, and uric acid crystals etc. [14]. Some important DAMPs and their receptors are mentioned in Table 1. Here, we review systematically the release and involvement of DAMPs in the aSAH pathophysiology. 

## 2. Methods

For retrieval of potential articles to be included in this review, we searched Pubmed using ‘Subarachnoid hemorrhage’ as a MeSH term in combination with other words in any field including ‘DAMP’, ‘Damage associated molecular pattern’, ‘Danger associated molecular pattern’, ‘alarmins’, HMGB1, S100B, Hemoglobin, Hb, Oxyhemoglobin, methemoglobin, heme, hemin, fibrinogen, fibronectin, extracellular matrix, ECM, Tenascin-C, IL-33, IL-1α, mitochondrial DNA, mtDNA, heat shock protein, HSP etc. Based on the above mentioned criteria, 829 total articles were found. After excluding duplicates and also selecting the relevant references from the retrieved articles, finally 134 publications were selected to review the role of DAMPs in aSAH. The search of relevant articles was performed by SRC and SM.

## 3. Results

### 3.1. High Mobility Group Box 1 (HMGB1) and aSAH

HMGB1 is a well characterized prototypical protein DAMP. HMGB1 is expressed in all eukaryotic cells as a non-histone DNA binding nuclear transcription factor, but signifies danger upon its extracellular release from necrotic cells [21]. Extracellularly released HMGB1 is then recognized by Toll Like Receptor (TLR)-2, TLR-4, TLR-9, and receptor for advanced glycation end products (RAGE) [21]. Evidence on the role of HMGB1 after aSAH is increasing continuously in the recent years. Release of HMGB1 in the cerebrospinal fluid of patients after aSAH was first found by Nakahara et al., (2009). Interestingly, the elevated HMGB1 levels were higher in the cerebrospinal fluid (CSF) of patients with a poor clinical outcome after aSAH and HMGB1 levels correlated with TNF-α, IL-6, and IL-8, suggesting an indispensable role of HMGB1 in ongoing inflammation [22]. King and colleagues also found significant associations of CSF HMGB1 levels with poor Hunt and Hess (H&H) grades and the disability and dependence among aSAH patients [23]. A subsequent study employing a rabbit model of SAH has shown that HMGB1 was upregulated and translocated in the cytosol of the microglia for active secretion [24]. Zhu, et al. [25] evaluated HMGB1 levels in systemic circulation and demonstrated an association with CVS, poor functional outcomes, and mortality after one year of aSAH, highlighting the prognostic value of on admission plasma HMGB1 determination. 

Sun, et al. [26] found as early as 2 h post SAH release of HMGB1 from the neurons and intraventricular injection of recombinant HMGB1 upregulated the inflammation as assessed by upregulation of TLR-4, NF-κB, IL-1β, and cleaved Caspase-3. Furthermore, in-vitro application of hemoglobin (Hb) led to the upregulation and translocation of HMGB1 from nucleus to cytoplasm in neuronal cultures. Interestingly, application of Glycyrrhizic acid, a natural inhibitor of HMGB1, downregulated IL-1β and thus, prevented activation of glial cells upon conditioned medium application from Hb primed neurons [26]. Thereafter, two other natural compounds, Purpurogallin, a natural phenol and 4′-*O*-β-d-glucosyl-5-*O*-methylvisamminol were demonstrated to attenuate HMGB1 expression in double hemorrhagic SAH rat model, and intriguingly, were also effective in decreasing the cerebral vasospasm and its associated changes in basilar arteries [27,28]. A similar study employing Rhinacanthin-C, an extract from *Rhinacanthus nasutus,* ameliorated SAH associated increase in HMGB1 mRNA and protein as well as pro-inflammatory cytokines and cleavage of Caspase-3 and Caspase 9 [29].

Another clinical study described elevated CSF HMGB1 levels in acute hydrocephalus after aSAH and strong correlations with H&H score, World Federation of Neurological Surgeons (WFNS) score, Glasgow Coma Scale (GCS), and days on intensive care unit and poor outcome after 3 months [30]. Wang and colleagues confirmed the association of CSF HMGB1 levels and poor outcome after 3 months in a relatively larger cohort of aSAH patients. Further, they revealed that in the SAH rat model both HMGB1 and its receptor RAGE are upregulated and application of post SAH CSF either from patients or rats induced RAGE expression and reduced viability of neuronal cultures. Interestingly, administration of the recombinant soluble form of RAGE to interfere with RAGE and HMGB1 signaling reduced the neuronal cell death both in-vitro and in-vivo [31]. The first evidence that HMGB1 may be involved in the inflammatory response leading to CVS, the most feared complication after aSAH, came from the observations of Zhao and colleagues. They observed increased expression of HMGB1 in the vasospastic rat basilar arteries at day 3, 5, and 7 after SAH [32]. Li, et al. [33] have shown increased basilar artery thickness and reduced luminal diameter with increased expression of HMGB1 protein and mRNA of pro-inflammatory cytokines, and all these changes were ameliorated after glycyrrhizic acid supplementation for 3 days [33]. Finally, administration of anti-HMGB1 antibody prevented basilar artery vasospasm, decreased extracellular translocation, and expression of HMGB1 in smooth muscle cells, decreased the expression of contractile and inflammation associated molecules, decreased plasma HMGB1 levels, improved the morphology and decreased the number of cerebral cortex microglia, and lastly, recovery from the neurological deficits [34].

DCI, the main cause of secondary decline in patients with aSAH, is seen in approximately 30% of the patients [35]. A case series of three aSAH patients with DCI has shown significant elevation of HMGB1 compared to controls, but did not show significant changes in both CSF and plasma HMGB1 levels as compared to baseline. Interestingly, there was a trend towards increase in plasma and decrease in CSF HMGB1 levels [36]. Moreover, the presence of minor allele G of rs2249825 has been found to be an independent predictor of DCI. This single nucleotide polymorphism (SNP) of HMGB1 (C/G at 3814) may lead to enhanced HMGB1 expression and consequently may result in DCI [37]. The above discussed evidence suggests that HMGB1 not only plays a distinct role during early brain injury, but also in post aSAH sequelae with prominent involvement in CVS, DCI, and thereby may impact the clinical outcome. Pharmacological strategies to neutralize HMGB1 might have therapeutic potential to improve the clinical outcome after aSAH.

### 3.2. S100B

S100 proteins, also termed calgranulins, are intracellular small calcium binding proteins and consist of more than 20 members [38]. S100A8, S100A9, and S100A12 are expressed by phagocytes [39] and the first identified member of this class, S100B is majorly expressed in the brain by astrocytes, although some neuronal populations also express it [40]. Passively released S100B by necrotic and damaged cells, has diagnostic and prognostic value in different CNS pathologies including traumatic brain injury when present above threshold limits in the CSF, serum or amniotic fluid [41]. At higher micromolar concentrations, extracellular S100B behaves as DAMP with neurotoxic effects mediated by RAGE and is involved in many neurodegenerative and inflammatory brain diseases [42]. It can induce neuronal death, expression of pro-inflammatory cytokines such as IL-1β and stress related inflammatory enzymes such as inducible nitric oxide synthase (iNOS) [43].

S100 protein levels have been shown to significantly rise after aSAH and correlate with severity of aSAH and peak after the onset of vasospasm [44]. Persson et al., have shown elevated S100 levels in CSF of SAH patients and Hardemark et al., observed elevated S100 levels were associated with H&H grade, degree of blood on CT, functional outcome assessed by GOS, and various neuropsychological evaluation tests [45,46]. Systemic levels of S100B were elevated at admission, 3 days and 7 days after aSAH and were associated with poor H&H grade and clinical outcome (GOS score) after 6 months [47]. Later on, Kay and colleagues, while investigating ApoE, incidentally found significantly higher CSF S100B levels after aSAH [48]. Serial determination of CSF from aSAH patients showed elevated levels of S100B, which correlated strongly with τ (Tau) protein CSF levels and clinical outcome after 3 months [49]. A significant rise of CSF S100B on admission followed by a gradual decrease over six days and a subsequent delayed elevation in serum was observed in SAH patients [50]. Interestingly, a second non-significant peak of S100B occurred at Day 4 of determination, probably reflecting secondary brain injury due to vasospasm [50]. 

Brain extracellular fluid (ECF) S100B levels determination in a case of aSAH by microdialysis showed an initial S100B peak after SAH and further, two sharp peaks in S100B levels occurred during the periods of vasospasms [51]. Lefranc et al. [52], observed a highly significant increase in S100B, S100A2, and other S100 analogs during vasospasm in a rat double hemorrhagic SAH model [52]. Furthermore, it has been demonstrated that an increased expression of S100B correlated with protein kinase C-η (eta) in the endothelial cells and protein kinase C-ζ (zeta) in the smooth muscle cells of the vasospastic basilar arteries after SAH [53]. 

Furthermore, WFNS scores, daily mean value of S100B above a threshold of 0.4 µg/L, and age independently predicted poor outcome at 6 months after aSAH and S100B time course was observed to be higher in patients undergoing neurosurgical clipping, having higher WFNS score and Fischer score [54]. A subsequent study found association of on admission serum S100B with WFNS score, Fischer score, and one year outcome, and on admission S100B above a threshold of 0.3 µg/L predicted poor clinical outcome and short term survival [55]. S100B elevation also reflected secondary brain deterioration due to vasospasm induced ischemia, brain edema, and hydrocephalus [55]. Hydrocephalus and elevated mean 8-day S100B levels predict poor clinical outcome one year after aSAH [56]. Another study evaluated mean 15-day S100B levels as an independent predictor of poor clinical outcome after one year with a better cut off value of 0.23 µg/L associated with a sensitivity of 91% and specificity of 90% [57]. The patients experiencing vasospasm leading to cerebral ischemia had higher S100B levels from the beginning and enhanced incidence of poor outcome [57]. Kaneda, et al. [58] showed significant elevation of CSF S100B at day 3 and 14, Glial Fibrillary Acidic Protein (GFAP) at day 3 and 7, Malondialdehyde (MDA) at day 14 in poor outcome aSAH patients as markers of brain damage and oxidative stress [58]. Moritz, et al. [59] have shown the value of serial CSF and serum S100B determination as prognostic marker for cerebral infarction, intracranial hypertension and outcome at ICU discharge. 

Another study investigated impaired passage of S100B from CSF to serum by employing S100B serum/CSF ratio and albumin CSF/serum ratio and found S100B release in the periphery was independent of BBB dysfunction. Furthermore, a higher S100B serum/CSF ratio associated with better neurological function highlight a repair role for active stimulated release of S100B [60]. Piazza, et al. [61] proposed S100B as a link between brain and lung and its implication in neurogenic pulmonary edema after aSAH [61]. In post aSAH hydrocephalus, high CSF and serum S100B levels for 10 days correlate with ventriculoperitoneal shunt placement [62]. Serum S100B levels were shown to be significantly lower 24 h after operation in patients receiving magnesium sulphate, thus highlighting its prognostic potential [63]. Administration of atorvastatin reduced serum S100B levels among high WFNS score patients and reduced the severity and incidence of vasospasms leading to ischemia [64].

Interestingly, serum S100B levels have been shown to correlate not only with ischemia, rather with the size of ischemic lesion also, irrespective of treatment or CVS association [65]. A small study showed initial lower levels of serum and CSF S100B in patients who later on developed vasospasm with non-significant elevation of serum S100B only [66]. In one patient, who was confirmed for vasospasm and developed cerebral infarction, S100B levels showed a consequent peak [66]. In severe grade aSAH, peak serum S100B levels measured over 15 days were associated with mortality [67] which was in agreement with a previous study from Oertel, et al. [68], but noticeably, Oertel and colleagues have reported higher serum S100B in patients who did not develop vasospasm [68]. Kellermann et al., have shown association of both serum and CSF S100B with clinical outcome after 6 months and serum S100B > 0.7 µg/L with mortality and increment in CSF after EVD (external ventricular drain) exchange [69]. Azurmendi and coauthors described plasma S100B as a marker of long term (1 year) outcome prediction in aSAH patients and suggested a cutoff value of 0.2 µg/L as a superior discriminator at day 10 among good and poor outcome patients [70]. A pooled analysis by Lai and Du [71] also demonstrated a strong association with cerebral infarction and long term outcome, and not with angiographic vasospasm. However, interestingly, intracerebroventricular infusion of S100B in rats inhibited the neuronal and endothelial dependent vasodilation and this effect was abolished with soluble RAGE (sRAGE) [72]. These lines of evidence clearly highlight the role of S100B in aSAH pathophysiology.

### 3.3. Hemoglobin and Its Derivatives

The extravasated blood and its degradation products acutely trigger neuroinflammation in addition to global ischemic insult in aSAH [5,73]. Erythrocyte hemolysate degradation yields methemoglobin, heme, hemin, and oxyhemoglobin, which are described as TLR-4 receptor ligands and as DAMPs [74,75,76,77]. Methemoglobin, owing to its water solubility, may lead to widespread TLR-4 activation via enhanced CSF distribution remotely from the site of release and has been shown to correlate with TLR-4 activation product Tumor necrosis factor (TNF)-α [75]. Methemoglobin can also bind TLR-4/TLR-2 heterodimer in addition to TLR-4 homodimers [75]. Heme not only activates TLR-4, but also promotes increased formation of Neutrophil Extracellular Traps (NETs) from neutrophils [77]. Heme ligation of TLR-4 only activates Myeloid differentiation primary response protein 88 (MyD88) dependent substream pathway leading to NFκB and Mitogen activated protein kinase (MAPK) activation with resultant TNF-α secretion [78]. Hemin (iron (III)-protoporphyrin IX) acts additively to endotoxin with mechanism of TLR-4 activation distinct to endotoxin [74]. Oxyhemoglobin can induce TLR-4 expression and activation in vascular smooth muscle cells (VSMCs) paralleled by TNF-α secretion, but this effect might stem from spontaneous oxidation to methemoglobin [76,79].

It has already been well established that the degree of bleeding on initial computerized tomography (CT) scan correlates with poor clinical outcome [9]. It was also recognized a long time ago that hemoglobin and its derivatives released after erythrocyte hemolysis can induce contraction of cerebral arteries both in vitro and in vivo (reviewed in detail by [80]). Induction of basilar artery vasospasm by application of either Lipopolysaccharide (LPS)—a TLR-4 agonist or blood in the subarachnoid space indicated a common shared pathway upregulating inflammation after SAH [81]. Kwon et al. [75], have shown that the methemoglobin led to activation of microglia and macrophages, and TNF-α secretion in a TLR-4 dependent manner. Furthermore, intra-subarachnoid administration of methemoglobin activated microglia with enhanced TNF-α and TLR-4 upregulation [75]. Wu et al. [79], have previously demonstrated upregulation and activation of TLR-4 in VSMCs by oxyhemoglobin that could be modulated by PPAR-γ agonist Rosiglitazone.

Heme has demonstrated cytotoxic effects on macrophages, microglia, astrocytes, and brain endothelial cells [78]. Cell-free Hb binds plasmatic protein haptoglobin, while heme binds hemopexin and the resulting complexes are scavenged by Cluster of differentiation (CD)163 and CD91 on monocytes/macrophages, respectively, and results in hemeoxygenase-1 (HO-1) upregulation. HO-1 then metabolizes them to Carbon monoxide (CO), iron, and biliverdin which can modulate monocyte/macrophage polarization [82]. Interestingly, hemopexin injection is neuroprotective in cerebral ischemia model and hemopexin knockout mice are more susceptible to neuronal injury after intracerebral bleeding [83,84]. It has also been shown that heme has the potential to induce IL-1β secretion via Nucleotide-binding oligomerization domain, Leucine-rich repeat, and Pyrin domain containing protein 3 (NLRP3) inflammasome in macrophages [85]. However, in a rat filament model of SAH, it was shown that heme upregulated the expression of HO-1 around the hemorrhage site and IL-1α, which was confirmed in-vitro by application of heme to organotypic slice cultures preferentially releasing IL-1α over IL-1β [86].

Thrombolytic agents clearing intra subarachnoid clot has shown decrease in vasospasm and improvement in outcome [73]. Interestingly, cisternal irrigation with plasminogen activator in patients undergoing neurosurgical clipping have shown reduced serum inflammatory cytokines, reduced ischemic lesions and better clinical outcome [73]. Haptoglobin polymorphism has shown that Hp1-1 genotype has more anti-inflammatory and vasodilatory potential compared to Hp 2-1 or Hp 2-2, which might result from better Hb clearance, prostaglandins synthesis inhibition, better extravascular distribution, and ROS scavenging by Hp 1-1 [5,87]. Since, iron released from heme can generate toxic radicals via Fenton reaction, administration of Deferoxamine in SAH has shown reduced injury [78]. So, hemoglobin and its degradation products act as DAMPs and strategies aimed at their early removal or neutralization will potentially help to reduce the pathophysiological events triggered by subarachnoid blood hemolysis to prevent complications and improve clinical outcome. 

### 3.4. Fibrinogen

Fibrinogen, a 340 kDa plasma protein, is also implicated in the extravasated blood induced inflammation and is converted into fibrin on coagulation cascade activation [76]. Fibrin(ogen) is also released after extracellular matrix (ECM) cleavage [88]. Fibrinogen induces secretion of monocyte chemoattractant protein-1 (MCP-1), platelet derived growth factor-AB (PDGF-AB), and IL-8 in endothelial cells which lead to enhanced chemotaxis in monocytes in vitro [89]. In macrophages, fibrinogen induces increased mRNA expression of several chemokines including MCP-1 and increased secretion of MCP-1 in a TLR-4 dependent mechanism and reveals its role as a DAMP [90,91].

Extravascular fibrin leads to microglial activation and neuronal damage after stroke [92]. Fibrinogen mediated activation of microglia has been attributed to CD11b/CD18 dependent activation of Akt and Rho signaling, and neurite outgrowth inhibition to ligation of β3 integrins with consequent upregulation of neuronal Endothelial growth factor receptor (EGF-R) [93]. Transforming growth factor (TGF)-β and Smad signaling triggered by fibrinogen culminates in astrogliosis [94]. Cutting down the fibrin formation or abolishing its binding to the microglial CD11b/CD18 receptor has been shown to reduce clustering of perivascular microglia and axonal demise in EAE model [95]. Elevated blood fibrinogen levels have been shown to correlate with increased mortality long ago in SAH patients [96]. Increased CSF levels of fibrinogen and its degradation products have been seen after rebleeding and in patients presenting with vasospasm, severe neurological deficits, and cerebral ischemia after aSAH [97,98,99,100]. These findings highlight the role of fibrin(ogen) as a DAMP and its involvement in the ongoing neuroinflammation which requires further investigation in the context of aSAH.

### 3.5. IL-1α and IL-33

IL-1α and IL-33 are both members of the IL-1 family of cytokines and are synthesized as pro-forms requiring cleavage of around 100 amino acid residues at the N-terminal to give mature forms [101]. IL-1α and IL-33 share a unique feature that they have a dual role as intracellular transcriptional regulators and as extracellular potent regulators of inflammation [102]. Interestingly, both pro- and mature forms of IL-1α are active in inducing inflammation, whereas pro-IL-33 is not and probably requires processing by serine proteases extracellularly into its mature form [101]. IL-1α, signaling via IL-1R, is constitutively expressed in endothelial cells, keratinocytes, and fibroblasts, but in monocytes/macrophages its synthesis occurs de novo [102]. IL-1α binds not only to cellular receptors, but also functions as a transcription factor in the presence of pro-inflammatory stimuli such as LPS or TNF and promotes production of NF-κB (p65), IL-6, and IL-8 [103,104]. Moreover, IL-1α can mediate recruitment of neutrophils via increased secretion of CXCL-1 by mesenchymal cells [105].

As discussed above, in a rat filament model of SAH, IL-1α was expressed mainly in microglia/macrophages after 12 h with higher expression in basal structures adjacent to hemorrhage site in addition to cortex, striatum, and hippocampus and colocalize with HO-1 in activated microglia [86]. Moreover, application of heme upregulated the secretion of active form of IL-1α from organotypic slice cultures and mixed glial cell cultures and the administration of IL-1R antagonist reduced BBB breakdown and brain damage [86]. Interestingly, IL-1α gene expression was highest at day 7 and correlated with decreased vessel caliber in canine vasospastic basilar arteries isolated at different days after intracisternal blood injections [106]. Inhibition of p38-MAPK signaling reduced IL-1α gene and protein expression in human VSMCs in vitro and downregulated IL-1α mRNA expression in canine basilar arteries showing reversal of vasospasm [107]. Bowman, et al. [108] showed increased IL-1α levels in rat femoral arteries displaying vasospasm.

IL-33 is known to have anti-inflammatory activity via promoting Th2 type response. IL-33 can stimulate cells of innate and adaptive immunity via binding to Suppressor of Tumorigenicity (ST)2 membrane receptors [109]. ST2 receptor is a member of TLR/IL-1R superfamily and its heteromer with IL-1R accessory protein (IL-1RAcP) is responsible for IL-33 signaling, while soluble ST2 (sST2) act as a decoy receptor [110]. Interestingly, monocytes/macrophages are polarized towards alternate type (M2) phenotype in the presence of IL-33 [111]. However, IL-33 has been assigned to play an inflammatory role in CNS reflecting its pleiotropic nature [110,112]. Huang, et al. [113] have observed an increased expression of IL-33 mRNA and protein in the cerebral cortex of the rats after experimental SAH. Intriguingly, IL-33 expression colocalized with neuronal and astrocytic markers and mRNA expression of IL-33 correlated with that of IL-1β after SAH [113]. So, IL-1α and IL-33 represent important DAMPs implicated in neuroinflammation after experimental SAH and therefore, needs further investigations.

### 3.6. Mitochondrial DAMPs

In recent years, mitochondria have been recognized as a host of different DAMPs including mitochondrial transcription factor A (TFAM)), N-formyl peptides, cardiolipin, and hypomethylated/non-methylated mitochondrial DNA which are released upon cell stress, injury, and necrosis [114]. Mitochondrial DNA (mtDNA) has been identified a long time ago to induce TNF secretion from splenocytes and arthritis in mice joints [115]. Zhang, et al. [116] have shown that circulating mtDNA, acting via TLR-9, elicits MAPK-signaling based migration and degranulation of neutrophils leading to organ injury. There are now evidences that mtDNA can upregulate innate immune responses through several PRRs, most importantly TLR-9, NLRP3-, and NLR family CARD domain containing 4 (NLRC4)-. Absent in melanoma (AIM)2-inflammasome complex and cyclic GMP-AMP synthase—stimulator of Interferon genes (cGAS-STING) [117]. Several studies have shown elevated circulating cell-free mtDNA and its biomarker and prognostic potential in connection to diseases involving CNS pathology [118,119,120,121,122,123,124,125]. Wang and colleagues have evaluated plasma and CSF mtDNA levels from 21 aSAH patients and found significant elevation of mtDNA in the CSF on admission, which was associated with poor clinical outcome. However, plasma mtDNA levels showed a delayed elevation at day 8 in poor clinical outcome patients [126].

### 3.7. Extracellular Matrix Derived DAMPs

Components of the extracellular matrix (ECM), which are released upon proteolysis following tissue injury in soluble form can act as DAMPs [91]. These normally ECM sequestered components such as biglycan, decorin, versican, tenascin-C, hyaluronan, and heparan sulfate are recently recognized as rapid activators of innate immune response by interacting with PRRs after their release [91,127]. Hyaluronic acid has been shown to be increased after aSAH in CSF [128]. Bell and coauthors while investigating the role of glycocalyx in microthrombosis and inflammation leading to DCI after aSAH have found elevated plasma levels of syndecan-1 (SDC-1, a heparan sulfate proteoglycan) and sCD44 (hyaluronan receptor) implicating endothelial glycocalyx injury [36]. CSF levels of Tenascin-C have been shown to be associated with worse WFNS scores, shunt dependent chronic hydrocephalus, vasospasm, DCI, and clinical outcome after aSAH [129,130,131]. Tenascin-C was also shown to contribute towards BBB disruption, brain edema, and MAPK mediated upregulation of Matrix metalloproteinase (MMP)-9 and Zonaoccludens (ZO)-1 degradation [132]. A recent study investigated the involvement of periostin and tenascin-C in mediating early brain injury after experimental SAH in mice and antibody neutralization of periostin or genetic knockout of tenascin C provided improvement against brain injury and in neurobehavioural outcomes and decreased induction of each other [133]. 

Fibronectin, another ECM protein, activates TLR-2 and TLR-4 due to structural unfolding of its type III domains and involves NFκB and p38-MAPK downstream signaling [134]. Interestingly, lower levels of plasma fibronectin were found at day 3 and day 9 in patients with poor clinical outcome at 3 months and in those with vasospasm [135]. This observation coincided with low levels of fibronectin in rabbit basilar arteries harvested on day 3 showing vasospasm [136]. Galectin-3 (Gal-3), the only chimeric protein in galectin family with carbohydrate recognition domain and affinity for β-galactoside, serves dual function, acting not only as a PRR, but also as a DAMP [137]. Elevated plasma levels of Gal-3 were found to be associated with poor functional outcome and increased mortality along with poor WFNS and Fischer scores in aSAH [138].

### 3.8. Heat Shock Proteins 

Heat shock proteins (HSP) are highly conserved chaperones aiding in protein folding and represent another potential subgroup of DAMPs which can activate PRRs such as TLR-2 and TLR-4 leading to MyD88 dependent upregulation of NFκB [139]. Increased expression of HSP90α, HSP60, HSP27, and HSP10 has been shown in rat brain stem after SAH, but at protein level HSP10 and HSP27 were significantly expressed [140]. In rat SAH endovascular perforation model, HSP70 expression was induced in neurons and glia in multiple brain regions [141]. Application of lysed blood, whole blood, and oxyhemoglobin in subarachnoid space led to the upregulation of HSP32 (HO-1) expression in microglia throughout different brain regions, but another study showed increased HSP70, HSP32 expression in neurons, microglia, and astrocytes, while HSP47 only in microglia [142,143]. Impaired vasorelexation has been seen in the rat middle cerebral arteries after SAH and it has been attributed to upregulation of phosphorylated HSP27 and reduced expression of both total and phosphorylated HSP20 [144]. Interestingly, HSP72 has been shown to be upregulated during CVS and downregulation of HSP72 by antisense oligodeoxynucleotide aggravated, whereas its induction by geranylgeranylacetone (GGA) relieved CVS [145].

## 4. Discussion

DAMPs have been shown to be implicated in various CNS disorders, for instance, it has been demonstrated that HMGB1 is involved in ischemic/hypoxic and microhemorrhagic events occurring in the course of spontaneous seizures [146,147]. Furthermore, HMGB1 and its receptor RAGE have been shown to mediate the ischemic brain damage after stroke [148]. Since, DAMPs might be the initiators of inflammation and therefore, their early blockage or sequestration may be helpful to reduce the ongoing inflammation and reduce the severity of the disease with protection against ensuing complications and poor outcomes. HMGB1 represents an excellent example, where pre-clinical approaches to neutralize HMGB1 by administering anti-HMGB1 monoclonal antibody or other molecules (ethyl pyruvate, glycyrrhizic acid, ghrelin, purpurogallin, and siRNA) that inhibit the release of HMGB1 have been shown to be beneficial [26]. Recombinant soluble form of RAGE to interfere with RAGE and HMGB1 signaling has been shown to be neuroprotective in experimental SAH [31]. RAGE is implicated in the signaling of multiple DAMPs and represents a potential therapeutic target and similarly, sST-2 administration to abrogate IL-33 signaling in SAH needs to be investigated [149]. Quinolone-3-carboxamide can bind S100A9 and S100A8/S100A9 complex to inhibit interaction with TLR-4 and RAGE [149]. It has already been shown that mtDNA levels are upregulated after CNS insult and TLR-9 is the major receptor mediating inflammatory effects of mtDNA [150]. Therefore, TLR-9 represents an important target and different strategies based on oligodeoxynucleotides (ODN) aimed at antagonizing the inflammatory effects of TLR-9 activation are under development through preclinical or early clinical studies [151,152,153]. Another approach, based on molecular scavenging of the free nucleic acids by nuclear acid binding polymers, has been shown to limit the inflammation in preclinical studies [154,155]. Since toll-like receptors are implicated in the signaling of numerous DAMPs, therefore, they represent important modulatable targets to culminate DAMPs signaling during brain injury [156]. Furthermore, the other DAMPs receptors and the substream signaling pathways represent potential modulatable targets. 

Finally, investigation of the time course of various DAMPs may have a diagnostic and prognostic potential and will be helpful for early identification of the patients at increased risk of developing different complications and achieving poor clinical outcome. Therefore, it will aid in addressing early and aggressive treatment and management in these patients. Further, DAMPs may be used as treatment response markers. Systemic S100B and HMGB1 represent interesting DAMP molecules that have been investigated in aSAH associated complications and may serve as potential biomarkers. Our knowledge, regarding DAMPs and their implication in complex pathophysiological events triggered after brain injury, is still in infancy and further investigations aimed at combined multifaceted role of DAMPs in brain injury after aSAH are warranted.

## Figures and Tables

**Table 1:** List of important DAMPs members and their receptors.

Sr. #	DAMPs	Receptors
1.	HMGB1	TLR-2, TLR-4, TLR-9, RAGE
2.	IL-1α	IL-1R
3.	IL-33	ST2 (IL-1RL1)
4.	Heme, Hemin, Oxyhemoglobin, methemoglobin	TLR-4
5.	mtDNA	TLR-9, NLRP3, NLRC4, AIM-2, cGAS-STING
6.	TFAM	RAGE, TLR-9
7.	N-formyl peptides	FPR1, FPRL1
8.	S-100 proteins	TLR-4, RAGE
9.	Fibrinogen	TLR-4
10.	Fibronectin	TLR-2, TLR-4
11.	Hyaluronan	TLR-2, TLR-4
12.	Biglycan	TLR-2, TLR-4, P2X4, P2X7, NLRP3
13.	Versican	TLR-2, TLR-6, CD14
14.	Heparan sulfate	TLR-4
15.	Tenascin C	TLR-4
16.	Galectin-3	TLR-2, TLR-4

## Abbreviations

DAMPs	Damage Associated Molecular Patterns
aSAH	Aneurysmal Subarachnoid Hemorrhage
IL-1	Interleukin-1
HMGB1	High Mobility Group Box-1
mtDNA	Mitochondrial DNA
DINDs	Delayed Ischemic Neurological Deficits
CVS	Cerebral Vasospasm
DCI	Delayed Cerebral Ischemia
HSPs	Heat Shock Proteins
PRRs	Pattern Recognition Receptors
S100B	S100 Calcium Binding Protein Beta
ECM	Extracellular Matrix
RAGE	Receptor for Advanced Glycation Endproducts
CSF	Cerebrospinal Fluid
BBB	Blood Brain Barrier
TNF-α	Tumor Necrosis Factor-alpha
NF-κB	Nuclear Factor-κB
TLR-4	Toll Like Receptor-4
H&H	Hunt and Hess score
WFNS	World Federation of Neurological Surgeons score
GCS	Glasgow Coma Scale
GOS	Glasgow Outcome Scale
mRS	Modified Rankin Scale
GFAP	Glial Fibrillary Acidic Protein
CNS	Central Nervous System
EVD	External Ventricular Drain
iNOS	Inducible Nitric Oxide Synthase
Hb	Hemoglobin
PPAR-γ	Peroxisome Proliferator Activated Receptor-γ
Hp	Haptoglobin
HO-1	Heme Oxygenase-1
MDA	Malondialdehyde
MCP-1	Monocyte Chemoattractant Protein-1
PDGF-AB	Platelet Derived Growth Factor-AB
MAPK	Mitogen Activated Protein Kinase
LPS	Lipopolysaccharide
VSMCs	Vascular Smooth Muscle Cells
ODN	Oligodeoxynucleotides
Gal-3	Galectin-3
TFAM	Mitochondrial Transcription Factor A
EGF-R	Endothelial Growth Factor-Receptor
ST2	Suppressor of Tumorigenicity-2
MMP-9	Matrix Metalloproteinase-9
ZO-1	Zonaocculdens-1
SDC-1	Syndecan-1
GGA	Geranyl Geranyl Acetone
cGAS	cyclic GMP-AMP synthase
STING	Stimulator of Interferon Genes
NLRP3	Nucleotide-binding oligomerization domain, Leucine-rich repeat and Pyrin domain containing protein 3
NLRC4	NLR family CARD domain containing 4
AIM2	Absent in Melanoma 2

## References

[1] Zhao J., Lin H., Summers R., Yang M., Cousins B.G., Tsui J. (2018). Current treatment strategies for intracranial aneurysms: An overview. Angiology.

[2] Aoki T., Frȍsen J., Fukuda M., Bando K., Shioi G., Tsuji K., Ollikainen E., Nozaki K., Laakkonen J., Narumiya S. (2017). Prostaglandin E2–EP2–NF-κB signaling in macrophages as a potential therapeutic target for intracranial aneurysms. Sci. Signal..

[3] Etminan N., Rinkel G.J. (2016). Unruptured intracranial aneurysms: Development, rupture and preventive management. Nat. Rev. Neurol..

[4] Grasso G., Alafaci C., Macdonald R.L. (2017). Management of aneurysmal subarachnoid hemorrhage: State of the art and future perspectives. Surg. Neurol. Int..

[5] Macdonald R.L. (2014). Delayed neurological deterioration after subarachnoid haemorrhage. Nat. Rev. Neurol..

[6] Van Gijn J., Kerr R.S., Rinkel G.J.E. (2007). Subarachnoid haemorrhage. Lancet.

[7] De Rooij N.K., Linn F.H., van der Plas J.A., Algra A., Rinkel G.J. (2007). Incidence of subarachnoid haemorrhage: A systematic review with emphasis on region, age, gender and time trends. J. Neurol. Neurosurg. Psychiatry.

[8] Korja M., Kaprio J. (2016). Controversies in epidemiology of intracranial aneurysms and SAH. Nat. Rev. Neurol..

[9] Suarez J.I., Tarr R.W., Selman W.R. (2006). Aneurysmal subarachnoid hemorrhage. N. Engl. J. Med..

[10] Van Dijk B.J., Vergouwen M.D.I., Kelfkens M.M., Rinkel G.J.E., Hol E.M. (2016). Glial cell response after aneurysmal subarachnoid hemorrhage—Functional consequences and clinical implications. Biochim. Biophys. Acta (BBA) Mol. Basis Dis..

[11] Cahill J., Zhang J.H. (2009). Subarachnoid hemorrhage: Is it time for a new direction?. Stroke.

[12] Macdonald R.L., Schweizer T.A. (2017). Spontaneous subarachnoid haemorrhage. Lancet.

[13] Cahill J., Calvert J.W., Zhang J.H. (2006). Mechanisms of early brain injury after subarachnoid hemorrhage. J. Cereb. Blood Flow Metab..

[14] Chen G.Y., Nuñez G. (2010). Sterile inflammation: Sensing and reacting to damage. Nat. Rev. Immunol..

[15] Matzinger P. (1994). Tolerance, danger, and the extended family. Annu. Rev. Immunol..

[16] Takeuchi O., Akira S. (2010). Pattern recognition receptors and inflammation. Cell.

[17] Murray K.N., Parry-Jones A.R., Allan S.M. (2015). Interleukin-1 and acute brain injury. Front. Cell. Neurosci..

[18] Lucke-Wold B.P., Logsdon A.F., Manoranjan B., Turner R.C., McConnell E., Vates G.E., Huber J.D., Rosen C.L., Simard J.M. (2016). Aneurysmal subarachnoid hemorrhage and neuroinflammation: A comprehensive review. Int. J. Mol. Sci..

[19] Provencio J.J. (2013). Inflammation in subarachnoid hemorrhage and delayed deterioration associated with vasospasm: A review. Acta Neurochir. Suppl..

[20] O’Neill L.A.J., Golenbock D., Bowie A.G. (2013). The history of toll-like receptors—Redefining innate immunity. Nat. Rev. Immunol..

[21] Bianchi M.E., Manfredi A.A. (2009). Dangers in and out. Science.

[22] Nakahara T., Tsuruta R., Kaneko T., Yamashita S., Fujita M., Kasaoka S., Hashiguchi T., Suzuki M., Maruyama I., Maekawa T. (2009). High-mobility group box 1 protein in CSF of patients with subarachnoid hemorrhage. Neurocrit. Care.

[23] King M.D., Laird M.D., Sangeetha S.R., Youssef P., Shakir B., Vender J.R., Alleyne C.H., Dhandapani K.M. (2010). Elucidating novel mechanisms of brain injury following subarachnoid hemorrhage: An emerging role for neuroproteomics. Neurosurg. Focus.

[24] Murakami K., Koide M., Dumont T.M., Russell S.R., Tranmer B.I., Wellman G.C. (2011). Subarachnoid hemorrhage induces gliosis and increased expression of the pro-inflammatory cytokine high mobility group box 1 protein. Transl. Stroke Res..

[25] Zhu X.-D., Chen J.-S., Zhou F., Liu Q.-C., Chen G., Zhang J.-M. (2012). Relationship between plasma high mobility group box-1 protein levels and clinical outcomes of aneurysmal subarachnoid hemorrhage. J. Neuroinflamm..

[26] Sun Q., Wu W., Hu Y.C., Li H., Zhang D., Li S., Li W., Li W.D., Ma B., Zhu J.H. (2014). Early release of high-mobility group box 1 (HMGB1) from neurons in experimental subarachnoid hemorrhage in vivo and in vitro. J. Neuroinflamm..

[27] Chang C.-Z., Lin C.-L., Wu S.-C., Kwan A.-L. (2014). Purpurogallin, a natural phenol, attenuates high-mobility group box 1 in subarachnoid hemorrhage induced vasospasm in a rat model. Int. J. Vasc. Med..

[28] Chang C.-Z., Wu S.-C., Kwan A.-L., Lin C.-L. (2015). 4′-*O*-β-d-glucosyl-5-*O*-methylvisamminol, an active ingredient of *Saposhnikovia divaricata*, attenuates high-mobility group box 1 and subarachnoid hemorrhage-induced vasospasm in a rat model. Behav. Brain Funct..

[29] Chang C.Z., Wu S.C., Kwan A.L., Lin C.L. (2016). Rhinacanthin-C, a fat-soluble extract from *Rhinacanthus nasutus*, modulates high-mobility group box 1-related neuro-inflammation and subarachnoid hemorrhage-induced brain apoptosis in a rat model. World Neurosurg..

[30] Sokol B., Wozniak A., Jankowski R., Jurga S., Wasik N., Shahid H., Grzeskowiak B. (2015). HMGB1 level in cerebrospinal fluid as a marker of treatment outcome in patients with acute hydrocephalus following aneurysmal subarachnoid hemorrhage. J. Stroke Cerebrovasc. Dis..

[31] Wang K.-C., Tang S.-C., Lee J.-E., Li Y.-I., Huang Y.-S., Yang W.-S., Jeng J.-S., Arumugam T.V., Tu Y.-K. (2017). Cerebrospinal fluid high mobility group box 1 is associated with neuronal death in subarachnoid hemorrhage. J. Cereb. Blood Flow Metab..

[32] Zhao X.D., Mao H.Y., Lv J., Lu X.J. (2016). Expression of high-mobility group box-1 (HMGB1) in the basilar artery after experimental subarachnoid hemorrhage. J. Clin. Neurosci..

[33] Li Y., Sun F., Jing Z., Wang X., Hua X., Wan L. (2017). Glycyrrhizic acid exerts anti-inflammatory effect to improve cerebral vasospasm secondary to subarachnoid hemorrhage in a rat model. Neurol. Res..

[34] Haruma J., Teshigawara K., Hishikawa T., Wang D., Liu K., Wake H., Mori S., Takahashi H.K., Sugiu K., Date I. (2016). Anti-high mobility group box-1 (HMGB1) antibody attenuates delayed cerebral vasospasm and brain injury after subarachnoid hemorrhage in rats. Sci. Rep..

[35] Francoeur C.L., Mayer S.A. (2016). Management of delayed cerebral ischemia after subarachnoid hemorrhage. Crit. Care.

[36] Bell J.D., Rhind S.G., Di Battista A.P., Macdonald R.L., Baker A.J. (2017). Biomarkers of glycocalyx injury are associated with delayed cerebral ischemia following aneurysmal subarachnoid hemorrhage: A case series supporting a new hypothesis. Neurocrit. Care.

[37] Hendrix P., Foreman P.M., Harrigan M.R., Fisher W.S.R., Vyas N.A., Lipsky R.H., Lin M., Walters B.C., Tubbs R.S., Shoja M.M. (2017). Impact of high-mobility group box-1 polymorphism on delayed cerebral ischemia following aneurysmal subarachnoid hemorrhage. World Neurosurg..

[38] Bianchi M.E. (2007). DAMPs, PAMPs and alarmins: All we need to know about danger. J. Leukoc. Biol..

[39] Foell D., Wittkowski H., Vogl T., Roth J. (2007). S100 proteins expressed in phagocytes: A novel group of damage-associated molecular pattern molecules. J. Leukoc. Biol..

[40] Sorci G., Bianchi R., Riuzzi F., Tubaro C., Arcuri C., Giambanco I., Donato R. (2010). S100B protein, a damage-associated molecular pattern protein in the brain and heart, and beyond. Cardiovasc. Psychiatry Neurol..

[41] Sen J., Belli A. (2007). S100B in neuropathologic states: The CRP of the brain?. J. Neurosci. Res..

[42] Bianchi R., Kastrisianaki E., Giambanco I., Donato R. (2011). S100B protein stimulates microglia migration via rage-dependent up-regulation of chemokine expression and release. J. Biol. Chem..

[43] Huang M., Dong X.Q., Hu Y.Y., Yu W.H., Zhang Z.Y. (2010). High S100B levels in cerebrospinal fluid and peripheral blood of patients with acute basal ganglial hemorrhage are associated with poor outcome. World J. Emerg. Med..

[44] Takayasu M., Shibuya M., Kanamori M., Suzuki Y., Ogura K., Kageyama N., Umekawa H., Hidaka H. (1985). S-100 protein and calmodulin levels in cerebrospinal fluid after subarachnoid hemorrhage. J. Neurosurg..

[45] Hardemark H.G., Almqvist O., Johansson T., Pahlman S., Persson L. (1989). S-100 protein in cerebrospinal fluid after aneurysmal subarachnoid haemorrhage: Relation to functional outcome, late CT and SPECT changes, and signs of higher cortical dysfunction. Acta Neurochir..

[46] Persson L., Hårdemark H.G., Gustafsson J., Rundström G., Mendel-Hartvig I., Esscher T., Påhlman S. (1987). S-100 protein and neuron-specific enolase in cerebrospinal fluid and serum: Markers of cell damage in human central nervous system. Stroke.

[47] Wiesmann M., Missler U., Hagenstrom H., Gottmann D. (1997). S-100 protein plasma levels after aneurysmal subarachnoid haemorrhage. Acta Neurochir..

[48] Kay A., Petzold A., Kerr M., Keir G., Thompson E., Nicoll J. (2003). Temporal alterations in cerebrospinal fluid amyloid beta-protein and apolipoprotein e after subarachnoid hemorrhage. Stroke.

[49] Kay A., Petzold A., Kerr M., Keir G., Thompson E., Nicoll J. (2003). Decreased cerebrospinal fluid apolipoprotein E after subarachnoid hemorrhage: Correlation with injury severity and clinical outcome. Stroke.

[50] Petzold A., Keir G., Lim D., Smith M., Thompson E.J. (2003). Cerebrospinal fluid (CSF) and serum S100B: Release and wash-out pattern. Brain Res. Bull..

[51] Sen J., Belli A., Petzold A., Russo S., Keir G., Thompson E.J., Smith M., Kitchen N. (2005). Extracellular fluid S100B in the injured brain: A future surrogate marker of acute brain injury?. Acta Neurochir..

[52] Lefranc F., Golzarian J., Chevalier C., DeWitte O., Pochet R., Heizman C., Decaestecker C., Brotchi J., Salmon I., Kiss R. (2002). Expression of members of the calcium-binding S-100 protein family in a rat model of cerebral basilar artery vasospasm. J. Neurosurg..

[53] Lefranc F., Decaestecker C., Brotchi J., Heizmann C.W., Dewitte O., Kiss R., Mijatovic T. (2005). Co-expression/co-location of S100 proteins (S100B, S100A1 and S100A2) and protein kinase C (PKC-beta, -eta and -zeta) in a rat model of cerebral basilar artery vasospasm. Neuropathol. Appl. Neurobiol..

[54] Weiss N., Sanchez-Pena P., Roche S., Beaudeux J.L., Colonne C., Coriat P., Puybasset L. (2006). Prognosis value of plasma S100B protein levels after subarachnoid aneurysmal hemorrhage. Anesthesiology.

[55] Stranjalis G., Korfias S., Psachoulia C., Kouyialis A., Sakas D.E., Mendelow A.D. (2007). The prognostic value of serum S-100B protein in spontaneous subarachnoid haemorrhage. Acta Neurochir..

[56] Pereira A.R., Sanchez-Pena P., Biondi A., Sourour N., Boch A.L., Colonne C., Lejean L., Abdennour L., Puybasset L. (2007). Predictors of 1-year outcome after coiling for poor-grade subarachnoid aneurysmal hemorrhage. Neurocrit. Care.

[57] Sanchez-Pena P., Pereira A.R., Sourour N.A., Biondi A., Lejean L., Colonne C., Boch A.L., Al Hawari M., Abdennour L., Puybasset L. (2008). S100B as an additional prognostic marker in subarachnoid aneurysmal hemorrhage. Crit. Care Med..

[58] Kaneda K., Fujita M., Yamashita S., Kaneko T., Kawamura Y., Izumi T., Tsuruta R., Kasaoka S., Maekawa T. (2010). Prognostic value of biochemical markers of brain damage and oxidative stress in post-surgical aneurysmal subarachnoid hemorrhage patients. Brain Res. Bull..

[59] Moritz S., Warnat J., Bele S., Graf B.M., Woertgen C. (2010). The prognostic value of NSE and S100B from serum and cerebrospinal fluid in patients with spontaneous subarachnoid hemorrhage. J. Neurosurg. Anesthesiol..

[60] Kleindienst A., Meissner S., Eyupoglu I.Y., Parsch H., Schmidt C., Buchfelder M. (2010). Dynamics of S100B release into serum and cerebrospinal fluid following acute brain injury. Acta Neurochir. Suppl..

[61] Piazza O., Venditto A., Tufano R. (2011). Neurogenic pulmonary edema in subarachnoid hemorrage. Panminerva Med..

[62] Brandner S., Xu Y., Schmidt C., Emtmann I., Buchfelder M., Kleindienst A., Schuhmann U.M., Czosnyka M. (2012). Shunt-dependent hydrocephalus following subarachnoid hemorrhage correlates with increased S100B levels in cerebrospinal fluid and serum. Intracranial Pressure and Brain Monitoring XIV.

[63] Hassan T., Nassar M., Elhadi S.M., Radi W.K. (2012). Effect of magnesium sulfate therapy on patients with aneurysmal subarachnoid hemorrhage using serum S100B protein as a prognostic marker. Neurosurg. Rev..

[64] Sanchez-Pena P., Nouet A., Clarencon F., Colonne C., Jean B., Le Jean L., Fonfrede M., Aout M., Vicaut E., Puybasset L. (2012). Atorvastatin decreases computed tomography and S100-assessed brain ischemia after subarachnoid aneurysmal hemorrhage: A comparative study. Crit. Care Med..

[65] Jung C.S., Lange B., Zimmermann M., Seifert V. (2013). CSF and serum biomarkers focusing on cerebral vasospasm and ischemia after subarachnoid hemorrhage. Stroke Res. Treat..

[66] Amiri M., Astrand R., Romner B. (2013). Can S100B predict cerebral vasospasms in patients suffering from subarachnoid hemorrhage?. Front. Neurol..

[67] De Azua Lopez Z.R., Egea-Guerrero J.J., Rivera-Rubiales G., Rodriguez-Rodriguez A., Vilches-Arenas A., Murillo-Cabezas F. (2015). Serum brain injury biomarkers as predictors of mortality after severe aneurysmal subarachnoid hemorrhage: Preliminary results. Clin. Chem. Lab. Med..

[68] Oertel M., Schumacher U., McArthur D.L., Kastner S., Boker D.K. (2006). S-100B and NSE: Markers of initial impact of subarachnoid haemorrhage and their relation to vasospasm and outcome. J. Clin. Neurosci..

[69] Kellermann I., Kleindienst A., Hore N., Buchfelder M., Brandner S. (2016). Early CSF and serum S100B concentrations for outcome prediction in traumatic brain injury and subarachnoid hemorrhage. Clin. Neurol. Neurosurg..

[70] Azurmendi L., Degos V., Tiberti N., Kapandji N., Sanchez-Pena P., Sarrafzadeh A., Puybasset L., Turck N., Sanchez J.C. (2016). Neopterin plasma concentrations in patients with aneurysmal subarachnoid hemorrhage: Correlation with infection and long-term outcome. J. Neurosurg..

[71] Lai P.M., Du R. (2016). Association between S100B levels and long-term outcome after aneurysmal subarachnoid hemorrhage: Systematic review and pooled analysis. PLoS ONE.

[72] Changyaleket B., Xu H., Vetri F., Valyi-Nagy T., Paisansathan C., Chong Z.Z., Pelligrino D.A., Testai F.D. (2016). Intracerebroventricular application of S100B selectively impairs pial arteriolar dilating function in rats. Brain Res..

[73] Miller B.A., Turan N., Chau M., Pradilla G. (2014). Inflammation, vasospasm, and brain injury after subarachnoid hemorrhage. BioMed Res. Int..

[74] Piazza M., Damore G., Costa B., Gioannini T.L., Weiss J.P., Peri F. (2011). Hemin and a metabolic derivative coprohemin modulate the TLR4 pathway differently through different molecular targets. Innate Immun..

[75] Kwon M.S., Woo S.K., Kurland D.B., Yoon S.H., Palmer A.F., Banerjee U., Iqbal S., Ivanova S., Gerzanich V., Simard J.M. (2015). Methemoglobin is an endogenous toll-like receptor 4 ligand-relevance to subarachnoid hemorrhage. Int. J. Mol. Sci..

[76] Kurland D.B., Gerzanich V., Simard J.M. (2015). DAMPs converging on toll-like receptor 4 in hemorrhagic stroke, a mini-review. Curr. Neurobiol..

[77] Gladwin M.T., Ofori-Acquah S.F. (2014). Erythroid damps drive inflammation in SCD. Blood.

[78] Dutra F.F., Bozza M.T. (2014). Heme on innate immunity and inflammation. Front. Pharmacol..

[79] Wu Y., Zhao X.D., Zhuang Z., Xue Y.J., Cheng H.L., Yin H.X., Shi J.X. (2010). Peroxisome proliferator-activated receptor gamma agonist rosiglitazone attenuates oxyhemoglobin-induced toll-like receptor 4 expression in vascular smooth muscle cells. Brain Res..

[80] Macdonald R.L., Weir B.K. (1991). A review of hemoglobin and the pathogenesis of cerebral vasospasm. Stroke.

[81] Recinos P.F., Pradilla G., Thai Q.-A., Perez M., Hdeib A.M., Tamargo R.J. (2006). Controlled release of lipopolysaccharide in the subarachnoid space of rabbits induces chronic vasospasm in the absence of blood. Surg. Neurol..

[82] Soares M.P., Bozza M.T. (2016). Red alert: Labile heme is an alarmin. Curr. Opin. Immunol..

[83] Dong B., Cai M., Fang Z., Wei H., Zhu F., Li G., Dong H., Xiong L. (2013). Hemopexin induces neuroprotection in the rat subjected to focal cerebral ischemia. BMC Neurosci..

[84] Chen L., Zhang X., Chen-Roetling J., Regan R.F. (2011). Increased striatal injury and behavioral deficits after intracerebral hemorrhage in hemopexin knockout mice. J. Neurosurg..

[85] Dutra F.F., Alves L.S., Rodrigues D., Fernandez P.L., de Oliveira R.B., Golenbock D.T., Zamboni D.S., Bozza M.T. (2014). Hemolysis-induced lethality involves inflammasome activation by heme. Proc. Natl. Acad. Sci. USA.

[86] Greenhalgh A.D., Brough D., Robinson E.M., Girard S., Rothwell N.J., Allan S.M. (2012). Interleukin-1 receptor antagonist is beneficial after subarachnoid haemorrhage in rat by blocking haem-driven inflammatory pathology. Dis. Model. Mech..

[87] Langlois M.R., Delanghe J.R. (1996). Biological and clinical significance of haptoglobin polymorphism in humans. Clin. Chem..

[88] Rosin D.L., Okusa M.D. (2011). Dangers within: DAMP responses to damage and cell death in kidney disease. J. Am. Soc. Nephrol..

[89] Seeger F.H., Blessing E., Gu L., Bornhold R., Denger S., Kreuzer J. (2002). Fibrinogen induces chemotactic activity in endothelial cells. Acta Physiol. Scand..

[90] Smiley S.T., King J.A., Hancock W.W. (2001). Fibrinogen stimulates macrophage chemokine secretion through toll-like receptor 4. J. Immunol..

[91] Schaefer L. (2014). Complexity of danger: The diverse nature of damage-associated molecular patterns. J. Biol. Chem..

[92] Adhami F., Liao G., Morozov Y.M., Schloemer A., Schmithorst V.J., Lorenz J.N., Dunn R.S., Vorhees C.V., Wills-Karp M., Degen J.L. (2006). Cerebral ischemia-hypoxia induces intravascular coagulation and autophagy. Am. J. Pathol..

[93] Ryu J.K., Davalos D., Akassoglou K. (2009). Fibrinogen signal transduction in the nervous system. J. Thromb. Haemost. JTH.

[94] Schachtrup C., Ryu J.K., Helmrick M.J., Vagena E., Galanakis D.K., Degen J.L., Margolis R.U., Akassoglou K. (2010). Fibrinogen triggers astrocyte scar formation by promoting the availability of active TGF-beta after vascular damage. J. Neurosci..

[95] Davalos D., Ryu J.K., Merlini M., Baeten K.M., Le Moan N., Petersen M.A., Deerinck T.J., Smirnoff D.S., Bedard C., Hakozaki H. (2012). Fibrinogen-induced perivascular microglial clustering is required for the development of axonal damage in neuroinflammation. Nat. Commun..

[96] Ettinger M.G. (1970). Coagulation abnormalities in subarachnoid hemorrhage. Stroke.

[97] Fodstad H., Nilsson I.M. (1981). Coagulation and fibrinolysis in blood and cerebrospinal fluid after aneurysmal subarachnoid haemorrhage: Effect of tranexamic acid (AMCA). Acta Neurochir..

[98] Huh Y.D., Yim M.B., Son E.I., Kim D.W., Lee J.K., Kim I.H., Jeon D.S. (1990). Blood antithrombin III and cerebrospinal fluid fibrin/fibrinogen degradation products in aneurysmal subarachnoid hemorrhage patients. J. Korean Neurosurg. Soc..

[99] Schisano G., Franco A., Nina P., Papa M.L., Iannuzzi M., De Biase R., Caldora M. (1994). Monitoring of fibrin and fibrinogen degradation products (FDP) in the cerebrospinal fluid of patients with subarachnoid haemorrhage due to ruptured aneurysm. Report of 55 cases. J. Neurosurg. Sci..

[100] Van der Werf A.J. (1986). Vascular spasm and cerebral ischemia after meningeal hemorrhage caused by rupture of an aneurysm. Neuro-Chir..

[101] Kim B., Lee Y., Kim E., Kwak A., Ryoo S., Bae S.H., Azam T., Kim S., Dinarello C.A. (2013). The interleukin-1α precursor is biologically active and is likely a key alarmin in the IL-1 family of cytokines. Front. Immunol..

[102] Hirsiger S., Simmen H.-P., Werner C.M.L., Wanner G.A., Rittirsch D. (2012). Danger signals activating the immune response after trauma. Mediat. Inflamm..

[103] Buryskova M., Pospisek M., Grothey A., Simmet T., Burysek L. (2004). Intracellular interleukin-1alpha functionally interacts with histone acetyltransferase complexes. J. Biol. Chem..

[104] Werman A., Werman-Venkert R., White R., Lee J.K., Werman B., Krelin Y., Voronov E., Dinarello C.A., Apte R.N. (2004). The precursor form of IL-1alpha is an intracrine proinflammatory activator of transcription. Proc. Natl. Acad. Sci. USA.

[105] Eigenbrod T., Park J.H., Harder J., Iwakura Y., Nunez G. (2008). Cutting edge: Critical role for mesothelial cells in necrosis-induced inflammation through the recognition of IL-1 alpha released from dying cells. J. Immunol..

[106] Aihara Y., Kasuya H., Onda H., Hori T., Takeda J. (2001). Quantitative analysis of gene expressions related to inflammation in canine spastic artery after subarachnoid hemorrhage. Stroke.

[107] Sasaki T., Kasuya H., Onda H., Sasahara A., Goto S., Hori T., Inoue I. (2004). Role of p38 mitogen-activated protein kinase on cerebral vasospasm after subarachnoid hemorrhage. Stroke.

[108] Bowman G., Dixit S., Bonneau R.H., Chinchilli V.M., Cockroft K.M. (2004). Neutralizing antibody against interleukin-6 attenuates posthemorrhagic vasospasm in the rat femoral artery model. Neurosurgery.

[109] Chackerian A.A., Oldham E.R., Murphy E.E., Schmitz J., Pflanz S., Kastelein R.A. (2007). IL-1 receptor accessory protein and ST2 comprise the IL-33 receptor complex. J. Immunol..

[110] Jiang H.-R., Milovanović M., Allan D., Niedbala W., Besnard A.-G., Fukada S.Y., Alves-Filho J.C., Togbe D., Goodyear C.S., Linington C. (2012). IL-33 attenuates EAE by suppressing IL-17 and IFN-γ production and inducing alternatively activated macrophages. Eur. J. Immunol..

[111] Kurowska-Stolarska M., Stolarski B., Kewin P., Murphy G., Corrigan C.J., Ying S., Pitman N., Mirchandani A., Rana B., van Rooijen N. (2009). IL-33 amplifies the polarization of alternatively activated macrophages that contribute to airway inflammation. J. Immunol..

[112] Hudson C.A., Christophi G.P., Gruber R.C., Wilmore J.R., Lawrence D.A., Massa P.T. (2008). Induction of IL-33 expression and activity in central nervous system glia. J. Leukoc. Biol..

[113] Huang L.T., Li H., Sun Q., Liu M., Li W.D., Li S., Yu Z., Wei W.T., Hang C.H. (2015). IL-33 expression in the cerebral cortex following experimental subarachnoid hemorrhage in rats. Cell. Mol. Neurobiol..

[114] Galluzzi L., Kepp O., Kroemer G. (2012). Mitochondria: Master regulators of danger signalling. Nat. Rev. Mol. Cell Biol..

[115] Collins L.V., Hajizadeh S., Holme E., Jonsson I.M., Tarkowski A. (2004). Endogenously oxidized mitochondrial DNA induces in vivo and in vitro inflammatory responses. J. Leukoc. Biol..

[116] Zhang Q., Raoof M., Chen Y., Sumi Y., Sursal T., Junger W., Brohi K., Itagaki K., Hauser C.J. (2010). Circulating mitochondrial damps cause inflammatory responses to injury. Nature.

[117] West A.P., Shadel G.S. (2017). Mitochondrial DNA in innate immune responses and inflammatory pathology. Nat. Rev. Immunol..

[118] Lu C.H., Chang W.N., Tsai N.W., Chuang Y.C., Huang C.R., Wang H.C. (2010). The value of serial plasma nuclear and mitochondrial DNA levels in adult community-acquired bacterial meningitis. QJM Mon. J. Assoc. Phys..

[119] Mathew A., Lindsley T.A., Sheridan A., Bhoiwala D.L., Hushmendy S.F., Yager E.J., Ruggiero E.A., Crawford D.R. (2012). Degraded mitochondrial DNA is a newly identified subtype of the damage associated molecular pattern (DAMP) family and possible trigger of neurodegeneration. J. Alzheimers Dis..

[120] Perez-Santiago J., Schrier R.D., de Oliveira M.F., Gianella S., Var S.R., Day T.R., Ramirez-Gaona M., Suben J.D., Murrell B., Massanella M. (2016). Cell-free mitochondrial DNA in CSF is associated with early viral rebound, inflammation, and severity of neurocognitive deficits in HIV infection. J. Neurovirol..

[121] Podlesniy P., Figueiro-Silva J., Llado A., Antonell A., Sanchez-Valle R., Alcolea D., Lleo A., Molinuevo J.L., Serra N., Trullas R. (2013). Low cerebrospinal fluid concentration of mitochondrial DNA in preclinical alzheimer disease. Ann. Neurol..

[122] Podlesniy P., Llorens F., Golanska E., Sikorska B., Liberski P., Zerr I., Trullas R. (2016). Mitochondrial DNA differentiates alzheimer’s disease from Creutzfeldt-Jakob disease. Alzheimer Dement..

[123] Podlesniy P., Vilas D., Taylor P., Shaw L.M., Tolosa E., Trullas R. (2016). Mitochondrial DNA in CSF distinguishes LRRK2 from idiopathic Parkinson’s disease. Neurobiol. Dis..

[124] Sondheimer N., Zollo O., Van Deerlin V., Trojanowski J.Q. (2014). Analysis of cerebrospinal fluid mitochondrial DNA levels in Alzheimer disease. Ann. Neurol..

[125] Varhaug K.N., Vedeler C.A., Myhr K.M., Aarseth J.H., Tzoulis C., Bindoff L.A. (2017). Increased levels of cell-free mitochondrial DNA in the cerebrospinal fluid of patients with multiple sclerosis. Mitochondrion.

[126] Wang H.C., Yang T.M., Lin W.C., Lin Y.J., Tsai N.W., Liou C.W., Kwan A.L., Lu C.H. (2013). The value of serial plasma and cerebrospinal fluid nuclear and mitochondrial deoxyribonucleic acid levels in aneurysmal subarachnoid hemorrhage. J. Neurosurg..

[127] Moreth K., Iozzo R.V., Schaefer L. (2012). Small leucine-rich proteoglycans orchestrate receptor crosstalk during inflammation. Cell Cycle.

[128] Heula A.L., Sajanti J., Majamaa K. (2015). Glycosaminoglycans in subdural fluid and CSF after meningeal injury. Acta Neurochir..

[129] Suzuki H., Hasegawa Y., Kanamaru K., Zhang J.H. (2010). Mechanisms of osteopontin-induced stabilization of blood-brain barrier disruption after subarachnoid hemorrhage in rats. Stroke.

[130] Suzuki H., Kanamaru K., Shiba M., Fujimoto M., Kawakita F., Imanaka-Yoshida K., Yoshida T., Taki W. (2015). Tenascin-C is a possible mediator between initial brain injury and vasospasm-related and -unrelated delayed cerebral ischemia after aneurysmal subarachnoid hemorrhage. Acta Neurochir. Suppl..

[131] Suzuki H., Kinoshita N., Imanaka-Yoshida K., Yoshida T., Taki W. (2008). Cerebrospinal fluid tenascin-C increases preceding the development of chronic shunt-dependent hydrocephalus after subarachnoid hemorrhage. Stroke.

[132] Fujimoto M., Shiba M., Kawakita F., Liu L., Shimojo N., Imanaka-Yoshida K., Yoshida T., Suzuki H. (2016). Deficiency of tenascin-C and attenuation of blood-brain barrier disruption following experimental subarachnoid hemorrhage in mice. J. Neurosurg..

[133] Liu L., Kawakita F., Fujimoto M., Nakano F., Imanaka-Yoshida K., Yoshida T., Suzuki H. (2017). Role of periostin in early brain injury after subarachnoid hemorrhage in mice. Stroke.

[134] Kelsh R., You R., Horzempa C., Zheng M., McKeown-Longo P.J. (2014). Regulation of the innate immune response by fibronectin: Synergism between the III-1 and EDA domains. PLoS ONE.

[135] Kashiwagi S., Shiroyama Y., Iwamoto T., Yamashita T., Ito H. (1993). Sequential changes in plasma fibronectin in patients with subarachnoid hemorrhage. Neurol. Medico-Chir..

[136] Kurogi R., Kikkawa Y., Matsuo S., Nakamizo A., Mizoguchi M., Sasaki T. (2015). Upregulation of tissue inhibitor of metalloproteinase-1 contributes to restoration of the extracellular matrix in the rabbit basilar artery during cerebral vasospasm after subarachnoid hemorrhage. Brain Res..

[137] Diaz-Alvarez L., Ortega E. (2017). The many roles of galectin-3, a multifaceted molecule, in innate immune responses against pathogens. Mediat. Inflamm..

[138] Liu H., Liu Y., Zhao J., Liu H., He S. (2016). Prognostic value of plasma galectin-3 levels after aneurysmal subarachnoid hemorrhage. Brain Behav..

[139] Kang J.-W., Kim S.-J., Cho H.-I., Lee S.-M. (2015). Damps activating innate immune responses in sepsis. Ageing Res. Rev..

[140] Satoh M., Tang J., Nanda A., Zhang J.H. (2003). Heat shock proteins expression in brain stem after subarachnoid hemorrhage in rats. Acta Neurochir. Suppl..

[141] Matz P.G., Sundaresan S., Sharp F.R., Weinstein P.R. (1996). Induction of HSP70 in rat brain following subarachnoid hemorrhage produced by endovascular perforation. J. Neurosurg..

[142] Matz P., Turner C., Weinstein P.R., Massa S.M., Panter S.S., Sharp F.R. (1996). Heme-oxygenase-1 induction in glia throughout rat brain following experimental subarachnoid hemorrhage. Brain Res..

[143] Turner C.P., Panter S.S., Sharp F.R. (1999). Anti-oxidants prevent focal rat brain injury as assessed by induction of heat shock proteins (HSP70, HO-1/HSP32, HSP47) following subarachnoid injections of lysed blood. Mol. Brain Res..

[144] Macomson S.D., Brophy C.M., Miller W., Harris V.A., Shaver E.G. (2002). Heat shock protein expression in cerebral vessels after subarachnoid hemorrhage. Neurosurgery.

[145] Nikaido H., Tsunoda H., Nishimura Y., Kirino T., Tanaka T. (2004). Potential role for heat shock protein 72 in antagonizing cerebral vasospasm after rat subarachnoid hemorrhage. Circulation.

[146] Gualtieri F., Marinelli C., Longo D., Pugnaghi M., Nichelli P.F., Meletti S., Biagini G. (2013). Hypoxia markers are expressed in interneurons exposed to recurrent seizures. Neuromol. Med..

[147] Lucchi C., Vinet J., Meletti S., Biagini G. (2015). Ischemic–hypoxic mechanisms leading to hippocampal dysfunction as a consequence of status epilepticus. Epilepsy Behav..

[148] Muhammad S., Barakat W., Stoyanov S., Murikinati S., Yang H., Tracey K.J., Bendszus M., Rossetti G., Nawroth P.P., Bierhaus A. (2008). The hmgb1 receptor rage mediates ischemic brain damage. J. Neurosci..

[149] Boyapati R.K., Rossi A.G., Satsangi J., Ho G.T. (2016). Gut mucosal DAMPs in IBD: From mechanisms to therapeutic implications. Mucosal Immunol..

[150] Boyapati R.K., Tamborska A., Dorward D.A., Ho G.-T. (2017). Advances in the understanding of mitochondrial DNA as a pathogenic factor in inflammatory diseases. F1000Research.

[151] Hennessy E.J., Parker A.E., O’Neill L.A.J. (2010). Targeting toll-like receptors: Emerging therapeutics?. Nat. Rev. Drug Discov..

[152] Savva A., Roger T. (2013). Targeting toll-like receptors: Promising therapeutic strategies for the management of sepsis-associated pathology and infectious diseases. Front. Immunol..

[153] Hoque R., Farooq A., Malik A., Trawick B.N., Berberich D.W., McClurg J.P., Galen K.P., Mehal W. (2013). A novel small molecule enantiomeric analogue of traditional (−)-morphinans has specific TLR9 antagonist properties and reduces sterile inflammation induced organ damage. J. Immunol..

[154] Holl E.K., Shumansky K.L., Borst L.B., Burnette A.D., Sample C.J., Ramsburg E.A., Sullenger B.A. (2016). Scavenging nucleic acid debris to combat autoimmunity and infectious disease. Proc. Natl. Acad. Sci. USA.

[155] Holl E.K., Shumansky K.L., Pitoc G., Ramsburg E., Sullenger B.A. (2013). Nucleic acid scavenging polymers inhibit extracellular DNA-mediated innate immune activation without inhibiting anti-viral responses. PLoS ONE.

[156] Downes C.E., Crack P.J. (2010). Neural injury following stroke: Are toll-like receptors the link between the immune system and the CNS?. Br. J. Pharmacol..

